# Comparative Effectiveness of Tricyclic Antidepressants, Serotonin–Norepinephrine Reuptake Inhibitors, and Anticonvulsants for Diabetic Painful Neuropathy: A Systematic Review and Meta-Analysis

**DOI:** 10.3390/medsci14060649

**Published:** 2026-10-08

**Authors:** Sreeya Reddy, Shakeel Ahmed, Munmun Chattopadhyay

**Affiliations:** 1 Paul L. Foster School of Medicine, Texas Tech Health El Paso, El Paso, TX 79905, USA; sreeredd@ttep.edu; 2 Department of Molecular and Translational Medicine, Paul L. Foster School of Medicine, Texas Tech Health El Paso, El Paso, TX 79905, USA; shakeel.ahmed@ttep.edu

**Keywords:** diabetic neuropathy, pain, amitriptyline, duloxetine, gabapentin, pregabalin

## Abstract

**Introduction:** Diabetic painful neuropathy (DPN) is one of the most common and debilitating complications of diabetes, substantially impairing the quality of life of patients. In the absence of disease-modifying therapies, treatment remains focused on symptomatic pain control with non-opioid pharmacologic agents; however, comparative evidence guiding optimal medication selection remains limited. Using a systematic review, pairwise meta-analysis, and network meta-analysis, this study compares the effectiveness of current non-opioid-based treatments, including amitriptyline, duloxetine, gabapentin, and pregabalin, for pain reduction in patients with DPN. **Methods:** PubMed and Google Scholar were systematically searched for randomized and clinical trials published between 2010 and 2025 evaluating these medications as monotherapy for DPN. A total of *n* = 11 studies met the inclusion criteria. Pain outcomes measured using the Numeric Rating Scale (NRS), Visual Analog Scale (VAS), or Brief Pain Inventory (BPI) were standardized to a common metric. Random-effects pairwise meta-analyses were performed to estimate within-drug treatment effects, followed by a frequentist network meta-analysis to compare therapies through direct and indirect evidence. Mean change in pain severity was the primary outcome. We performed heterogeneity, network consistency, and sensitivity analyses under varying correlation assumptions. **Results:** A total of 1397 participants across 11 studies were included. Pairwise meta-analysis demonstrated substantial pain reduction with duloxetine (mean change [MC] 27.8; 95% CI 18.5–37.1), gabapentin (MC 34.8; 95% CI 9.3–60.3), and pregabalin (MC 19.7; 95% CI 8.9–30.5). Although amitriptyline trials individually reported improvement, the pooled estimate did not reach statistical significance. Network meta-analysis indicated that gabapentin, duloxetine, and amitriptyline were associated with better estimated analgesic benefit than pregabalin; the differences among gabapentin, duloxetine, and amitriptyline were modest and not statistically significant. Sensitivity analyses supported the robustness of the findings, and no significant network inconsistency was detected. **Conclusions:** Commonly used non-opioid pharmacologic therapies for DPN provide broadly comparable analgesic benefit, with gabapentin showing comparatively preferable reduction in pain severity within the network analysis. Given the modest differences between agents and substantial variability in individual response, treatment decisions should be individualized according to patient comorbidities, tolerability, and therapeutic goals. Larger, methodologically standardized comparative trials are needed to strengthen the evidence base and support more precise, evidence-based management of DPN.

## 1. Introduction

Diabetic painful neuropathy (DPN) is a debilitating and widespread consequence of diabetes, affecting up to 50% of patients with long-standing disease [1]. DPN is a symmetric neuropathy that primarily affects the distal extremities and is associated with hyperalgesia and allodynia [2]. Characterized by burning, tingling, and numbness, DPN significantly impairs quality of life and increases the risk of foot ulcers, amputation, and healthcare utilization [3,4]. The pathogenesis of DPN is attributed to various factors that affect the Schwann cells lining peripheral nerves, such as non-enzymatic glycation, oxidative stress, and sorbitol accumulation [5]. In diabetes mellitus, the combined effects of this neuropathy and peripheral vascular ischemia impair both sensation and blood flow to the feet. As a result, small injuries often go unnoticed and fail to heal properly, predisposing patients to persistent ulcers and infections. Despite its high prevalence and significant clinical burden, current treatment options are primarily focused on symptom management [1]. There are currently no effective therapies that can prevent or reverse neuropathy or provide complete relief from neuropathic pain [6,7].

While glycemic control can delay progression, it does little to relieve established neuropathic pain [8]. To date, there are no proven disease-modifying therapies, and treatment is primarily focused on pain management [9]. Pharmacologic treatments such as opioids, tricyclic antidepressants (TCAs), serotonin–norepinephrine reuptake inhibitors (SNRIs), anticonvulsants (e.g., pregabalin, gabapentin), and topical capsaicin are commonly used for pain management [10]. However, recent comparative efficacy data are limited, and patient response varies widely [11]. Given the high prevalence of DPN, its profound impact on patient functioning, and the absence of curative therapies, optimizing pain management is a critical component of clinical care.

Previous systematic reviews and meta-analyses have examined the efficacy and safety of individual pharmacologic agents or different treatment classes for painful diabetic peripheral neuropathy (DPN), with many studies focusing on comparisons with placebo or evaluating individual treatments. The present analysis extends these studies by specifically comparing three commonly used non-opioid pharmacologic classes—SNRIs, TCAs, and anticonvulsants—for their effects on pain severity in adults with DPN. Unlike studies that evaluate individual or any combination treatments separately, our analysis compares these therapeutic classes to better understand differences in pain reduction. By focusing on comparative pain outcomes and including more recent evidence, this study addresses an important gap in the current literature and provides updated information that may help guide evidence-based and individualized treatment of painful DPN. Therefore, a clearer understanding of the relative effectiveness of commonly used non-opioid pharmacologic agents may support more personalized, evidence-based treatment strategies and ultimately improve outcomes for individuals living with diabetic painful neuropathy.

## 2. Materials and Methods

### 2.1. Data Source and Search Strategy

We systematically searched PubMed and Google Scholar for clinical trials evaluating amitriptyline, duloxetine, gabapentin, or pregabalin in adults with diabetic peripheral neuropathy. The search was conducted in September 2025 and limited to human studies and studies published from 1 January 2010 to 10 September 2025. The following search query was used on PubMed to screen eligible articles: (“Diabetic Neuropathies”[Mesh] OR “diabetic neuropathy”[tiab] OR “diabetic peripheral neuropathy”[tiab] OR “ diabetic painful neuropathy”[tiab]) AND (“Amitriptyline”[Mesh] OR amitriptyline[tiab] OR “Duloxetine Hydrochloride”[Mesh] OR duloxetine[tiab] OR “Gabapentin”[Mesh] OR gabapentin[tiab] OR “Pregabalin”[Mesh] OR pregabalin[tiab]) AND (“Clinical Trial”[pt] OR “Randomized Controlled Trial”[pt] OR randomized[tiab] OR placebo[tiab]). Google Scholar was searched in September 2025 using combinations of terms related to diabetic peripheral neuropathy and the target medications. Because Google Scholar produces extensive results that cannot be exhaustively screened, we reviewed the first 200 results sorted by relevance for each search string.

Abstracts and full-text articles were independently screened for eligibility by three reviewers (SR, MC, and SA). References cited in review articles were checked to ensure inclusion of any additional pertinent studies. We included any article evaluating the pain score reduction in patients with diabetic peripheral neuropathy after using amitriptyline, duloxetine, pregabalin, or gabapentin. Only studies published in English were considered eligible for inclusion. Descriptive studies (case series, case reports), non-human studies, abstracts, reviews, meta-analyses, and duplicate studies were not included in the review. This review was performed in accordance with the PRISMA (Preferred Reporting Items for Systematic Reviews and Meta-Analyses) guidelines (PRISMA 2020 checklist can be downloaded under Supplementary Materials).

### 2.2. Inclusion and Exclusion Criteria

We included studies (a) published from 2010 to the present that included adults who were diagnosed (b) with diabetic peripheral neuropathy, (c) having either type 1 or type 2 diabetes, (d) used amitriptyline, duloxetine, pregabalin, or gabapentin as monotherapy in the trial, (e) and reported pre- and post- or average change in pain using a Brief Pain Inventory (BPI), Numeric Rating Scale (NRS), or Visual Analog Scale (VAS). Only studies that reported sufficient data for network meta-analysis, i.e., mean change and standard deviation (SD); mean and SD for baseline and final; or median and quartiles for baseline and final, were included in the analysis. The study selection process is summarized in the PRISMA 2020 flow diagram (Figure 1).

### 2.3. Data Extraction and Synthesis

Each article was independently reviewed by three authors to reduce the risk of duplication and reporting bias. A structured data collection spreadsheet was created to systematically extract relevant study information. Specifically, we extracted the following key information from the eligible studies: study characteristics (title, last name of the first author, year of publication, sample size, the drug, dosage, duration of the study), type of pain scale used (BPI, NRS, or VAS), baseline demographics (gender ratio, mean age of participants, mean duration of DPN, mean HbA1c%, type of diabetes), and pain scale ratings. The primary outcome considered was pain reduction interpreted via the pain scales before and after therapy. To make scales consistent (all on 0–100), we converted the VAS from a 0–10 to 0–100 scale and the Norfolk-QOL-DN (NQD) score as *NQD_new_* = 100 × (*NQD_old_* + 4). This linear transformation onto a unified 100-point continuous metric aligns with standard Cochrane meta-analytic guidelines for continuous pain parameters, which recommend mapping diverse numeric scales onto a shared range to preserve absolute clinical interpretability and facilitate direct pooling without distorting individual trial effect sizes [12]. This linear rescaling approach has been empirically validated in large-scale clinical trial datasets, demonstrating that multiplying 10-point metrics up to a 100-point limit maintains strict statistical precision and introduces zero scale-conversion artifacts [13]. As a sensitivity analysis, all data points were linearly down-scaled to a unified 0–10 metric (dividing 0–100 values by 10), and the random-effects network meta-analysis was repeated across all active baseline drug references using identical frequentist graph-theoretical algorithms. For studies reporting pre-post medians and quartiles, these were approximated to means and SDs using the following relations [14]:

$$Mean=\frac{\left(Q1+Median+Q2\right)}{3}\;\;\;\;\mathrm{and}\;\;\;\;SD=\frac{\left(Q3-Q1\right)}{1.35}=\frac{QD}{1.35}$$

The mean change (MC) and SD of change for studies with pre- and post- statistics were obtained assuming a moderate choice of the correlation coefficient between pre- and post- data (i.e., ρ = 0.5) as follows:

$$MC={mean}_{pre}-{mean}_{post}$$

$$SD_{change}=\sqrt{{SD}_{pre}^{2}+{SD}_{post}^{2}-2\rho {SD}_{pre}{SD}_{post}}$$

The subscripts “pre” and “post” are used to denote the respective measures. Studies reporting post–pre mean changes were converted to pre–post differences to ensure comparability of statistics across all studies and drugs. A primary correlation of 0.5 was selected as a conservative, industry-standard default framework in accordance with the Cochrane Handbook for Systematic Reviews of Interventions, as it represents a statistically neutral midpoint that models moderate participant-level tracking across evaluation windows without introducing directional bias or artificially compressing the calculated within-study variance parameters.

### 2.4. Risk-of-Bias Assessment

Risk of bias in the included randomized trials was assessed using the revised Cochrane risk-of-bias tool for randomized trials (RoB 2) [15]. Each study was evaluated across five domains: bias arising from the randomization process, bias due to deviations from intended interventions, bias due to missing outcome data, bias in measurement of the outcome, and bias in selection of the reported result. Two reviewers independently assessed each study, and disagreements were resolved by consensus or consultation with a third reviewer. Study-level judgments were categorized as low risk, some concerns, or high risk. Risk-of-bias assessments are summarized in Supplementary Table S1.

### 2.5. Statistical Analysis

We conducted a descriptive analysis of the demographics reported across different studies. We then conducted a random-effects meta-analysis using the “netmeta” package in R [16] to pool results across studies for each drug. Pooled estimates were calculated using the inverse-variance weighting method, with between-study variance (τ^2^) estimated via the restricted maximum-likelihood (REML) approach. Untransformed (raw) means were analyzed. Confidence intervals for τ^2^ and τ were obtained using the Q-profile method, and the Hartung-Knapp adjustment was applied to address uncertainty in between-study heterogeneity estimates [17,18].

A frequentist graph-theoretical meta-analysis was performed to compare the effects of four treatments-amitriptyline, duloxetine, gabapentin, and pregabalin with six pairwise comparisons across four treatment designs, taking three drugs, pregabalin, duloxetine, and gabapentin, as reference categories. To strictly preserve within-study randomization and avoid the biases associated with naive pooling, the raw single-arm data (mean change and standard deviation of change) were transformed into contrast-based, relative treatment effects using the pairwise function. This function automatically matched treatment arms within each unique study identifier to calculate valid within-study Mean Differences (MD) and standard errors. A critical structural distinction was maintained between longitudinal pre–post analyses and these comparative network meta-analytic estimates. Raw pre–post metrics (the mean changes from baseline and their imputed standard deviations) track simple single-group within-arm longitudinal changes over time. Because these single-arm metrics do not possess an internal parallel control, they were never naively pooled across separate trial populations. Instead, the network meta-analysis model synthesized the study-specific relative contrasts exclusively, clearly separating single-group pre-post temporal tracking from true randomized relative treatment effects and ensuring that the global comparative estimates strictly preserve within-study randomized treatment allocation. The network meta-analysis was conducted using a graph-theoretical approach, enabling the simultaneous synthesis of direct and indirect evidence. Between-study variance (τ^2^) was estimated using the DerSimonian-Laird method [19]. Overall heterogeneity and inconsistency were evaluated using Cochran’s Q statistic, decomposed into within-design (heterogeneity) and between-design (inconsistency) components. The proportion of variability attributable to heterogeneity was quantified using the I^2^ statistic, calculated based on the Q statistic. To assess any significant bias due to the assumed correlation in missing studies, we performed a sensitivity analysis using correlation coefficients of 0.1 (low) and 0.9 (high).

Baseline demographics, including mean with SD for quantitative variables (e.g., age, HbA1c, duration of neuropathy) and percentages for categorical variables (e.g., sex), were calculated as weighted means, with sample size as the weight. This approach accounts for the fact that larger studies contribute more information and therefore prevents small studies from disproportionately influencing the overall estimates. These weighted demographic aggregates were further utilized to evaluate the clinical transitivity assumption across network nodes. Transitivity was evaluated descriptively by verifying that potential clinical and metabolic effect modifiers—including participant age, sex distribution, baseline HbA1c%, and disease duration—were uniformly distributed across all treatment comparison pathways, ensuring the populations were structurally comparable for simultaneous indirect synthesis. To address the impact of substantial pairwise heterogeneity and evaluate dosage disparities across the literature, a pre-specified subgroup sensitivity analysis was executed by restricting the network exclusively to trials evaluating guideline-recommended therapeutic maintenance doses of gabapentin (≥900 mg/day). Finally, to evaluate the mathematical validity of the network’s indirect routing and check for potential local discrepancies between direct and indirect evidence, a Separate Indirect from Direct Evidence (SIDE) node-splitting analysis was performed using the back-calculation method via the *netsplit()* function. Local inconsistency was evaluated for each closed loop, with statistical significance defined at a threshold of *p* < 0.05.

### 2.6. Publication Bias

Assessment of publication bias and small-study effects was conducted using comparison-adjusted funnel plots within the network meta-analysis framework. Funnel plots were generated under a moderate post-pre correlation assumption (ρ = 0.5), used to impute the standard deviation of change scores. To evaluate the robustness of findings to the choice of reference treatment, comparison-adjusted funnel plots were constructed using different drugs as the reference group (Pregabalin, Duloxetine, and Gabapentin). Asymmetry was checked visually. Formal statistical testing for small-study effects using Egger’s regression was performed at the pairwise meta-analysis level; however, given the limited number of studies per comparison, these results were interpreted cautiously and considered exploratory. Trim-and-fill analyses were conducted as an exploratory sensitivity analysis to assess the potential impact of unpublished studies on the pooled estimates. Although this method has limited reliability in the presence of substantial heterogeneity or few studies, it provides insight into the robustness of the findings to potential publication bias.

### 2.7. Registration and Protocol

This systematic review and meta-analysis were not registered, and no publicly available or formal protocol was prepared.

## 3. Results

### 3.1. Search Results

Our search yielded *n* = 396 articles from PubMed and Google Scholar. After screening and exclusion, only *n* = 11 met the eligibility criteria and were included in this systematic review and meta-analysis. Details of the search results are presented in the PRISMA flowchart.

### 3.2. Study Characteristics and Qualitative Synthesis

Several of the 11 included studies evaluated more than one pharmacologic agent. Valid outcome data (mean change and standard deviation of change for at least one of the three pain scales) were available for 3 amitriptyline, 6 duloxetine, 5 gabapentin, and 3 pregabalin study arms. Table 1 shows the characteristics of each of the studies included.

Among the baseline demographics, duloxetine contributed the largest evidence base (6 arms; *n* = 665), followed by pregabalin (3 arms; *n* = 307), gabapentin (5 arms; *n* = 224), and amitriptyline (3 arms; *n* = 201). Mean age was highest in the pregabalin group (60.96 ± 10.88 years) and lowest in the amitriptyline group (57.58 ± 10.31 years). Neuropathy duration was longest with pregabalin (4.52 years) and shortest with gabapentin (2.32 years), while HbA1c was highest in the gabapentin group (8.06%) and lowest in the pregabalin group (7.78%). The proportion of female participants was greatest in the gabapentin group (51.9%) and lowest in the amitriptyline group (35.5%). Only studies reporting a given variable were included in the calculation. In Table 2 below are the averaged baseline demographics for each drug group. An epidemiological appraisal of these aggregated study-level metrics confirmed that major potential clinical and metabolic effect modifiers were remarkably uniformly distributed across the treatment nodes. Specifically, mean patient age remained tightly clustered within a 3.3-year window across all groups (ranging from 57.58 ± 10.31 years to 60.96 ± 10.88 years), while glycemic control (mean baseline HbA1c) was virtually identical across cohorts, spanning a narrow range from 7.78% to 8.06%. While the mean duration of DPN showed a minor clinical shift for gabapentin (2.32 years) compared to pregabalin (4.52 years), all cohorts universally reflected well-established chronic states. This cross-study demographic balance satisfies the core clinical requirement of the transitivity assumption, ensuring that populations across the network remain structurally comparable for indirect synthesis. This qualitative evaluation was statistically reinforced by our loop-splitting diagnostic, which revealed zero significant local or global inconsistency across the network pathways (all *p* > 0.05).

### 3.3. Random-Effects Meta-Analysis: Pooled Drug Effects

A random-effects meta-analysis of n=1397 patients across four drug classes demonstrated statistically significant reductions in pain from baseline for three of the four drugs. The forest plot displays pooled mean changes using inverse-variance weighting (Figure 2).

For amitriptyline, although each individual trial showed a statistically significant improvement from baseline, the pooled random-effects estimate was not statistically significant (MC: 38.6; 95% CI: −11.8–89.0) as the 95% confidence interval (CI) includes zero (*p* > 0.05). This indicates that, when between-study variability is accounted for, the overall treatment effect is imprecise and could include no true benefit.

For duloxetine, the pooled random-effects estimate showed a statistically significant reduction in pain from baseline (MC: 27.8; 95% CI:18.5–37.1), displaying a 95% CI above zero (*p* < 0.05). This indicates a reliable overall treatment effect even after accounting for substantial heterogeneity across studies. Duloxetine also contributed the largest weight to the meta-analysis due to its larger combined sample size, making this estimate relatively more stable than for the other drugs. We observed similar improvement in pain score for gabapentin (MC: 34.8; 95% CI: 9.3–60.3) and pregabalin (MC:19.7; 95% CI: 8.9 to 30.5) in the pooled random-effects models despite observing a high heterogeneity (I^2^ > 80%) across studies.

For gabapentin, the pooled random-effects model demonstrated substantial improvement in pain, as the 95% confidence interval remained entirely above zero. However, the wide confidence interval and very high heterogeneity indicate that the magnitude of benefit varied considerably across studies.

### 3.4. Between-Drug Comparisons

The network meta-analysis provides direct estimates of how each drug compares with every other drug. The exact study-level relative data vectors generated by the contrast-based data step are summarized in Supplementary Table S7. This analytical dataset transparently documents the relative mean differences and standard errors for each randomized within-study contrast. When amitriptyline was compared with pregabalin, it showed a mean advantage of about 5.76 points, indicating it reduced pain by nearly 6 points more than pregabalin across the included studies. Duloxetine also outperformed pregabalin, with a MC of about 4.98 points (95% CI: 4.40–5.55; *p* < 0.0001), indicating a moderate but consistent benefit. Gabapentin showed the largest advantage over pregabalin, with an MC of approximately 6.82 points (95% CI: 2.55–11.08; *p* = 0.0017), making it the strongest performer relative to the weakest drug in the network. When the three higher-performing drugs were compared, the differences were smaller but still informative. Gabapentin reduced pain by about 1.84 points more than duloxetine and about 1.06 points more than amitriptyline, while duloxetine was about 0.78 points better than amitriptyline. Although the differences in MC among amitriptyline, duloxetine, and gabapentin were not statistically significant (*p* > 0.05), they were all in the same direction, with gabapentin consistently showing the largest estimated effect. Taken together, these point estimates indicate an overall ranking in which gabapentin has the greatest average pain-reducing effect, followed by duloxetine, then amitriptyline, with pregabalin performing worst. Tests of network consistency showed no evidence of disagreement between direct and indirect evidence (global Q = 2.02, *p* = 0.57; within-design Q = 1.25, *p* = 0.26), and heterogeneity within the network was negligible (τ^2^ = 0, I^2^ = 0%), indicating that the relative treatment effects were statistically coherent across the network. The relative effects between all treatment pairs from the network meta-analysis are summarized in Table 3. The structural architecture of the randomized evidence base and the direct head-to-head comparisons are visualized in the network plot (Figure 3). The network comprises a closed loop connecting amitriptyline, duloxetine, and gabapentin, with pregabalin serving as a well-connected reference node. As shown by the edge values, direct evidence for several comparisons remains sparse, with three treatment pairs (amitriptyline vs. pregabalin, amitriptyline vs. duloxetine, and amitriptyline vs. gabapentin) supported by only one or two primary trials.

The network meta-analysis provides direct and indirect estimates of how each drug compares with every other drug. When amitriptyline was compared with pregabalin, it showed a mean advantage of about 5.76 points (95% CI: 1.66–9.85; *p* = 0.0059), indicating it reduced pain by nearly 6 points more than pregabalin across the included studies. Duloxetine also outperformed pregabalin, with an MC of about 4.98 points (95% CI: 4.40–5.55; *p* < 0.0001), indicating a moderate but consistent benefit. Gabapentin showed the better advantage over pregabalin, with an MC of approximately 6.82 points (95% CI: 2.55–11.08; *p* = 0.0017), making it the agent with the highest estimated treatment effect relative to the numerically lowest-performing drug in the network.

When the three higher-ranked drugs were compared, the differences were smaller and statistically indistinguishable. Gabapentin reduced pain by about 1.84 points more than duloxetine and about 1.06 points more than amitriptyline, while duloxetine was about 0.78 points better than amitriptyline. Crucially, the differences in MC among amitriptyline, duloxetine, and gabapentin were not statistically significant (all *p* > 0.05). Therefore, while point estimates indicate a numerical trend where gabapentin has the highest estimated average pain-reducing effect, followed by duloxetine, then amitriptyline, their clinical efficacy remains broadly comparable. Tests of network consistency showed no evidence of disagreement between direct and indirect evidence (global Q = 2.02, *p* = 0.57; within-design Q = 1.25, *p* = 0.26). Although the global network model reported negligible heterogeneity (τ^2^ = 0, I^2^ = 0%), this is highly likely to be a mathematical artifact caused by a lack of statistical power and limited degrees of freedom due to the small number of studies per loop. This limited capacity to detect true clinical variations across trials is reflected in the extremely wide 95% confidence interval for the network I^2^ (0.0% to 84.7%). The relative effects between all treatment pairs from the network meta-analysis are summarized in Table 3. To further verify local loop stability and evaluate the specific pathways contributing to each comparison, a formal loop-splitting validation using the SIDE approach was executed (Supplementary Table S5). The node-splitting diagnostic revealed zero statistically significant disagreement between direct trial findings and indirect calculations across all evaluated pathways (all *p* > 0.05), mathematically confirming that the direct and indirect evidence streams are in complete alignment and free from structural contradiction.

The results of the sensitivity analyses, summarized in Table 4, demonstrate that the direction and magnitude of the pooled effects remained consistent across assumptions, including scenarios with extremely low or extremely high pre-post correlations, indicating that the findings are robust to the choice of correlation.

When the network meta-analysis was restricted exclusively to trials evaluating guideline-recommended therapeutic maintenance doses of gabapentin (≥900 mg/day), the global model mathematically yielded a network variance of zero ($I^{2}$ = 0%, Q = 0.48, *p* = 0.7860; Supplementary Table S2). However, this absolute reduction in estimated heterogeneity must be interpreted with extreme caution, as it is heavily driven by a lack of statistical power and restricted degrees of freedom resulting from the reduction in sample size down to only four eligible studies (k = 4), rather than reflecting true clinical uniformity. Crucially, in this therapeutic maintenance cohort (k = 4), gabapentin’s treatment effect relative to pregabalin fell to 4.69 and lost statistical significance (*p* = 0.089). Furthermore, gabapentin shifted to the numerically lowest-ranked active agent, falling behind both duloxetine (MD: 4.98, *p* < 0.0001) and amitriptyline (MD: 4.91, *p* = 0.025). Head-to-head comparisons among the active maintenance therapies were highly non-significant (gabapentin vs. duloxetine MD: −0.28, *p* = 0.918; gabapentin vs. amitriptyline MD: −0.21, *p* = 0.933) (Supplementary Table S3). This mathematically demonstrates that the apparent superiority of gabapentin in the global model was heavily driven by sub-therapeutic dosing outliers, requiring extreme caution in clinical ranking interpretations.

When restricted strictly to pure unidimensional metrics (VAS/NRS), the overall treatment hierarchy remained identical to the primary model, with gabapentin maintaining the uppermost numerical point estimate relative to pregabalin (MD: 4.89), followed by amitriptyline (MD: 4.27) and duloxetine (MD: 2.72; Supplementary Table S4). However, the reduction in network size down to four studies (k = 4, o = 545) resulted in an absolute loss of statistical significance across all active comparisons, with all 95% confidence intervals crossing zero (amitriptyline *p* = 0.1250; duloxetine *p* = 0.3314; gabapentin *p* = 0.1288). Head-to-head comparisons among active interventions were similarly non-significant (gabapentin vs. duloxetine MD: 2.17, *p* = 0.3238; gabapentin vs. amitriptyline MD: −0.62, *p* = 0.7912). This indicates that while our directional therapeutic rankings are robust to instrument frameworks, absolute statistical precision is heavily limited by data sparsity when excluding composite scale data.

The network meta-analysis revealed no significant statistical heterogeneity ($I^{2}=0\%,\;\tau^{2}=0)$. While this suggests that the variation in treatment effects across the five included studies is attributable to sampling error rather than true between-study differences, this estimate should be interpreted with caution. The wide 95% confidence interval for $I^{2}$ (0.0% to 84.7%) indicates that the analysis may be underpowered to detect moderate-to-high heterogeneity. However, the lack of significant inconsistency between direct and indirect evidence (*p* = 0.7761) supports the internal validity of the network results.

### 3.5. Assessment of Publication Bias and Small-Study Effects

Comparison-adjusted funnel plots constructed under a moderate correlation assumption (ρ = 0.5) did not show substantial asymmetry, irrespective of the reference treatment used. Visual inspection of the funnel plots suggested a largely symmetrical distribution of studies around the comparison-adjusted pooled effects, indicating no strong evidence of publication bias or small-study effects. These patterns remained consistent across sensitivity analyses assuming low and high within-study correlations, supporting the robustness of the findings (Figure 4). The trim-and-fill procedure did not impute additional studies and resulted in minimal change to the pooled estimate, suggesting that the findings are robust to potential publication bias (Supplementary Figure S1).

## 4. Discussion

Diabetic painful neuropathy is highly prevalent, chronically disabling, and difficult to treat, with current therapies focused on symptom control rather than disease modification. DPN is not only a physical condition but is also strongly associated with higher rates of depression, anxiety, sleep disturbance, and reduced overall quality of life [31,32]. Chronic neuropathic pain can impair daily functioning, limit mobility, and increase emotional distress, creating a bidirectional relationship in which psychological comorbidities further exacerbate pain perception and disability. These broader consequences highlight that effective pain control has implications beyond symptom relief alone and underscore the importance of continued research to optimize therapeutic strategies for DPN to improve both physical and mental health outcomes.

### 4.1. Current Treatment Landscape and Prior Evidence

Several pharmacologic options are available for the management of DPN. Duloxetine, pregabalin, extended-release tapentadol, and a topical capsaicin formulation (8%) are currently approved by the FDA for this indication. However, a number of commonly used medications, including tricyclic antidepressants, serotonin–norepinephrine reuptake inhibitors, gabapentinoids, and sodium channel blockers, are frequently employed as first-line oral treatments despite lacking specific FDA approval for DPN [33]. Prior large clinical trials comparing agents such as amitriptyline, duloxetine, and pregabalin have reported no statistically significant differences in efficacy among the agents [21]. A large systematic review and meta-analysis by Finnerup et al. found that first-line agents for neuropathic pain, including tricyclic antidepressants, serotonin–norepinephrine reuptake inhibitors, gabapentin, and pregabalin, provide comparable pain relief on average, with no consistent evidence that any single drug has greater effects [12]. The authors also identified evidence of publication bias, suggesting that reported treatment effects may be modestly inflated due to the underrepresentation of small or negative studies. This highlights that apparent differences between agents in the literature may reflect publication bias rather than true differences in analgesic efficacy and underscores the value of comparative analyses that integrate both direct and indirect evidence.

### 4.2. Summary of Study Findings and Comparative Effectiveness

Based on this background, the present study synthesizes recent clinical trial data using both pairwise and network meta-analytic approaches to directly compare four commonly used pharmacologic treatments for diabetic painful neuropathy. By standardizing pain outcomes across studies, our analysis provides a comparative ranking of treatment effects, demonstrating that gabapentin, duloxetine, and amitriptyline had better outcome scores compared to pregabalin on average, with gabapentin showing the marginal projected benefit. These findings provide quantitative context for existing guideline recommendations and clarify relative treatment performance, which may offer clinicians additional evidence to select the initial choice of drug in routine practice.

Although the differences in mean change among the top three drug classes were minimal, with gabapentin showing the better reduction in pain, followed by duloxetine and amitriptyline, these differences can be clinically meaningful in the context of chronic neuropathic pain, where incremental reductions in pain intensity can translate into improved daily functioning and quality of life.

Although amitriptyline demonstrated statistically significant pain reduction in all the individual studies, the pooled random-effects estimate was not statistically significant. This phenomenon is expected when the number of contributing studies is small and between-study variability is high. Random-effects models incorporate heterogeneity into the variance estimate, increasing uncertainty and reducing precision, particularly in small meta-analyses [34]. In our analysis, the limited number of amitriptyline arms and substantial variability across study designs and populations likely contributed to an imprecise pooled estimate despite consistent within-study improvement [35]. In contrast, the pooled effects for gabapentin, duloxetine, and pregabalin were consistent with their individual statistically significant effects because variability in effect sizes across studies was lower than the overall treatment effect, allowing pooled estimates to retain adequate precision under random-effects modeling.

This pairwise imprecision stands in notable contrast to our global network framework, where amitriptyline achieved a precise, significant advantage over pregabalin. This apparent paradox represents a classic network meta-analysis attribute known as ‘borrowing strength’ through indirect pathways. Because direct head-to-head trials evaluating amitriptyline are historically sparse, individual pairwise comparisons suffer from severe power constraints and wide confidence intervals. However, within the interconnected frequentist network matrix, the global model mathematically synthesizes the sparse direct evidence with robust indirect evidence routed through mutually shared anchors (duloxetine and gabapentin). This multi-pronged indirect data structure dramatically reduces the pooled standard error, narrowing the 95% confidence intervals to uncover stable, statistically significant differences. The internal mathematical validity of this borrowing mechanism was thoroughly verified via our SIDE node-splitting diagnostic, which revealed zero significant local or global inconsistency across any network loops, confirming that the direct trial data and indirect calculations are in complete statistical alignment.

### 4.3. Mechanistic Interpretation of Treatment Effects

Mechanistically, the four agents evaluated target neuropathic pain through distinct pathways, which may contribute to differences in both the magnitude and consistency of analgesic responses. Gabapentin and pregabalin are antiepileptic agents that bind voltage-gated calcium channels in the central nervous system, reducing calcium-dependent release of excitatory neurotransmitters and dampening central sensitization [36]. Duloxetine, a serotonin–norepinephrine reuptake inhibitor, and amitriptyline, a tricyclic antidepressant, exert their analgesic effects primarily by increasing spinal cord noradrenaline levels and activating descending inhibitory pain pathways. Increases in serotonin and dopamine may further enhance these effects [37]. The relative ranking observed in this study may therefore reflect differences in how these agents modulate pain pathways, as well as differences in tolerability, achievable dose, and study design.

### 4.4. Pain Measurement and Outcome Assessment

Accurate interpretation of these findings depends in part on how pain was measured across studies. Pain is inherently subjective and influenced by emotional and sensory factors, making consistent measurement challenging. Validated instruments such as the Visual Analog Scale (VAS), Numeric Rating Scale (NRS), and Brief Pain Inventory (BPI) translate subjective pain into quantifiable outcomes, enabling comparison across studies. Although all three assess pain intensity, they differ in structure, sensitivity, and the extent to which they capture functional impact, which has implications for comparing effect sizes.

The NRS is an 11-point scale (0–10) and has demonstrated greater sensitivity and higher patient compliance compared with VAS and verbal rating scales [38,39]. The VAS consists of a horizontal line anchored by “no pain” and “worst imaginable pain” and is commonly reported on a 0–100 scale, though some studies rescale it to 0–10 to match NRS [40]. VAS scores correlate strongly with NRS, supporting their comparability [41]. The BPI assesses both pain severity and pain-related interference with daily functioning, including activity, mood, sleep, and enjoyment of life [42]. For this analysis, only the BPI-Severity score was used, as it is conceptually aligned with VAS and NRS and is specifically intended to quantify pain intensity rather than functional impairment [43]. The severity score is calculated as the average of four items: worst, least, average, and current pain.

Across DPN trials, pain is typically measured at baseline and follow-up timepoints, and we analyzed the mean change from baseline or derived it when not directly reported. While combining these instruments is defensible given their shared focus on pain intensity, careful standardization is necessary. Differences in measurement precision and dimensional scope mean that effect sizes are not perfectly interchangeable; unidimensional scales such as VAS or NRS may show larger apparent reductions than multidimensional instruments like the BPI, which capture broader functional burden. This distinction is particularly relevant in DPN, where pain is chronic, variable, and closely linked to sensory loss and disability.

### 4.5. Limitations

This study has a number of limitations. First, the overall number of eligible studies and total sample size were relatively small, particularly within individual treatment comparisons, which limited statistical power and reduced the precision of pooled and network estimates. Second, substantial heterogeneity was observed across several analyses. This variability likely reflects differences in pain assessment methods and outcome definitions across studies, particularly given the subjective nature of neuropathic pain reporting. Specifically, our reliance on raw Mean Differences across linearly harmonized metrics assumes clinical interchangeability between unidimensional metrics and multidimensional composite subscales. While standardizing these tools via a native Standardized Mean Difference framework is theoretically preferable to evaluate scale comparability, a standardized network calculation was mathematically precluded by data sparsity and incomplete variance reporting in the primary literature, forcing the exclusion of multiple trials and generating severe network inconsistency. Additional heterogeneity may have arisen from variation in study design, clinical setting, treatment duration, dosing and titration strategies, and baseline patient characteristics. Crucially, while our global network model exhibited no statistically significant inconsistency between direct and indirect pathways, this statistical alignment does not, by itself, guarantee true clinical transitivity. Variances in baseline neuropathy duration, target treatment dosages, and trial environments remain relevant potential effect modifiers across the literature. Consequently, the validity of our indirect comparisons remains inherently constrained by these underlying clinical diversities, and the resulting cross-drug hierarchical rankings must be interpreted as tentative clinical trends rather than absolute, uniform certainties. The interpretation of pooled estimates is limited by risk-of-bias concerns in several included studies, particularly related to incomplete reporting of randomization procedures, open-label study designs, missing outcome data, and reliance on subjective pain outcomes, which may affect the certainty of comparative treatment estimates. Third, diabetes type was incompletely and inconsistently reported across the included studies. Most trials enrolled mixed or broadly defined diabetic neuropathy populations, several enrolled only type 2 diabetes participants, and one did not clearly specify diabetes type. Only three studies provided overall type 1/type 2 diabetes distribution, and none reported treatment response stratified by diabetes type (Supplementary Table S6). As a result, differences in disease duration, glycemic control, metabolic characteristics, and neuropathy progression may have influenced treatment response and contributed to between-study variability. Finally, the limited number of available studies precluded robust subgroup and sensitivity analyses, including analyses restricted to more homogeneous populations or standardized treatment durations. Consequently, our ability to identify specific clinical or methodological sources of heterogeneity and to draw more definitive comparative conclusions was constrained.

### 4.6. Implications for Future Research

Considering these limitations, future studies would benefit from greater standardization of pain assessment and reporting of outcomes. The use of a single, consistent pain scale across trials, along with uniform reporting of summary statistics (including mean change and standard deviation), would improve comparability between studies and facilitate more robust, reliable meta-analyses. Stratifying analyses by diabetes type, baseline pain severity, and relevant comorbidities, including depression and anxiety, would help clarify sources of heterogeneity and identify predictors of treatment response. Incorporating functional and quality-of-life outcomes alongside pain intensity, as well as evaluating long-term effectiveness and tolerability, could further inform individualized treatment selection and improve clinical decision-making in the management of DPN.

## 5. Conclusions

This systematic review and meta-analysis combine the available clinical trial evidence comparing commonly used non-opioid pharmacologic therapies for diabetic painful neuropathy and demonstrate that these agents provide broadly similar analgesic benefit overall, with no clear or consistently better option across studies. Although network estimates marginally favored gabapentin, the relatively small between-drug differences and variability in individual response highlight the importance of tailoring treatment decisions to patient-specific factors, including comorbidities, adverse-effect profiles, contraindications, tolerability, and therapeutic goals. These findings support a personalized approach to management while underscoring the limitations of the current evidence base. Future large-scale, methodologically standardized comparative trials are needed to better define relative efficacy and optimize treatment algorithms, ultimately improving pain control, functional outcomes, and quality of life for patients living with persistent neuropathic pain.

## Figures and Tables

**Figure 1:**
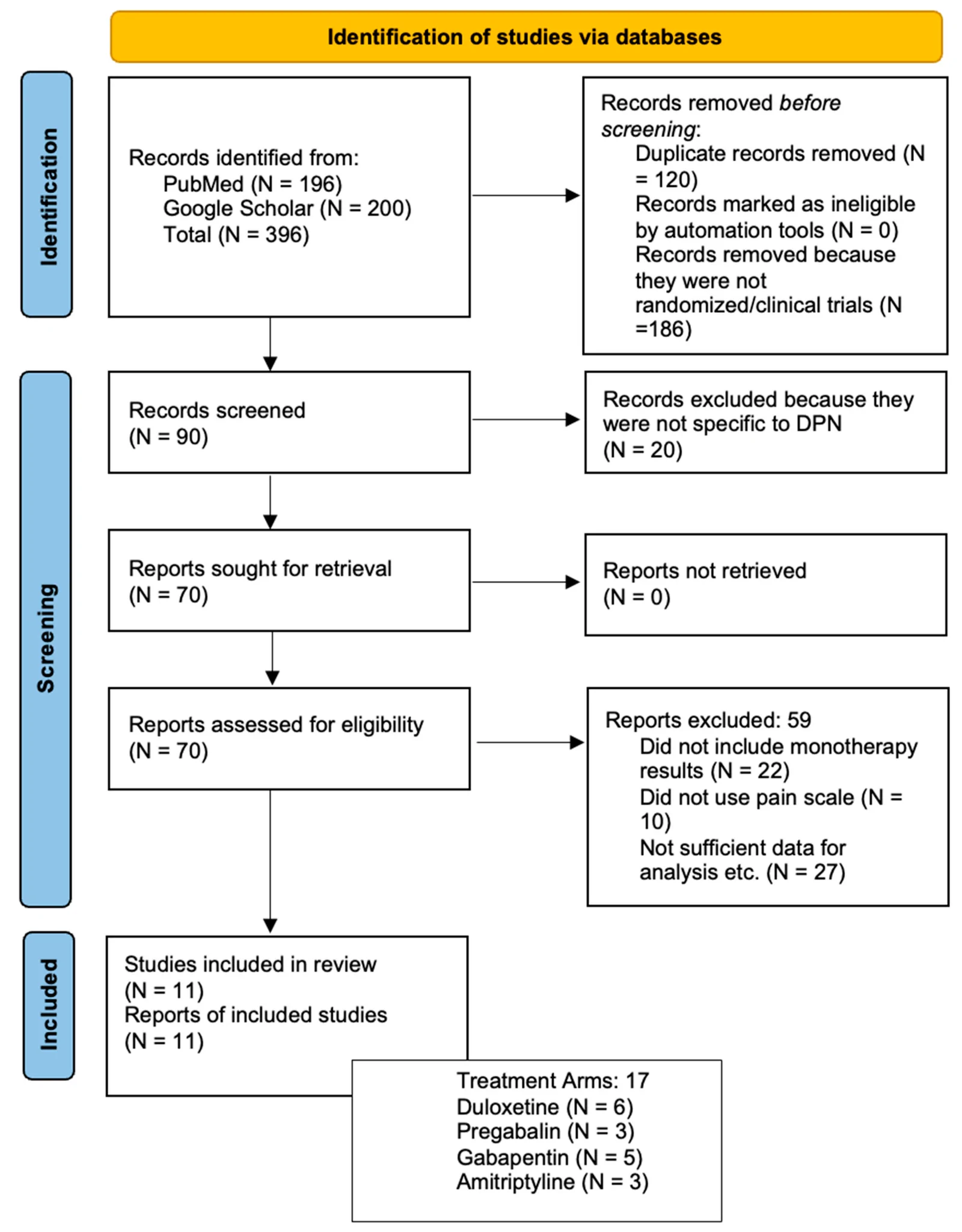
PRISMA 2020 flow diagram of study selection. DPN, Diabetic Painful Neuropathy. The *n* = 11 included studies evaluated multiple pharmacologic agents. Six studies included duloxetine, three included pregabalin, five included gabapentin, and three included amitriptyline; several studies evaluated more than one drug.

**Figure 2:**
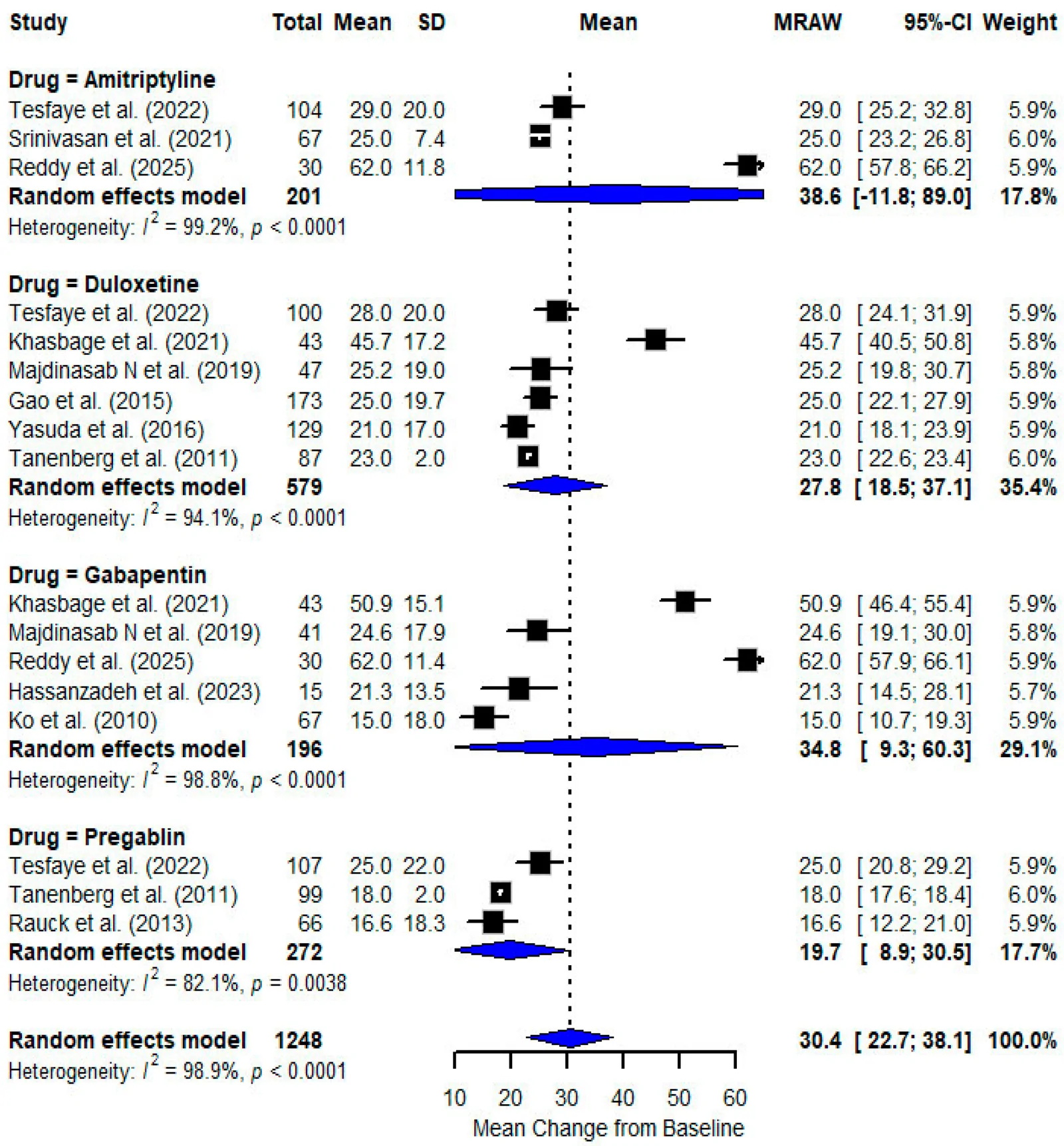
Pairwise random-effects meta-analysis of mean change in pain from baseline for each drug. References: Tesfaye et al. (2022) [20]; Srinivasan et al. (2021) [21]; Reddy et al. (2025) [22]; Khasbage et al. (2021) [23]; Majdinasab et al. (2019) [24]; Gao et al. (2015) [25]; Yasuda et al. (2016) [26]; Tanenberg et al. (2011) [27]; Hassanzadeh et al. (2023) [29]; Ko et al. (2010) [30]; Rauck et al. (2013) [28].

**Figure 3:**
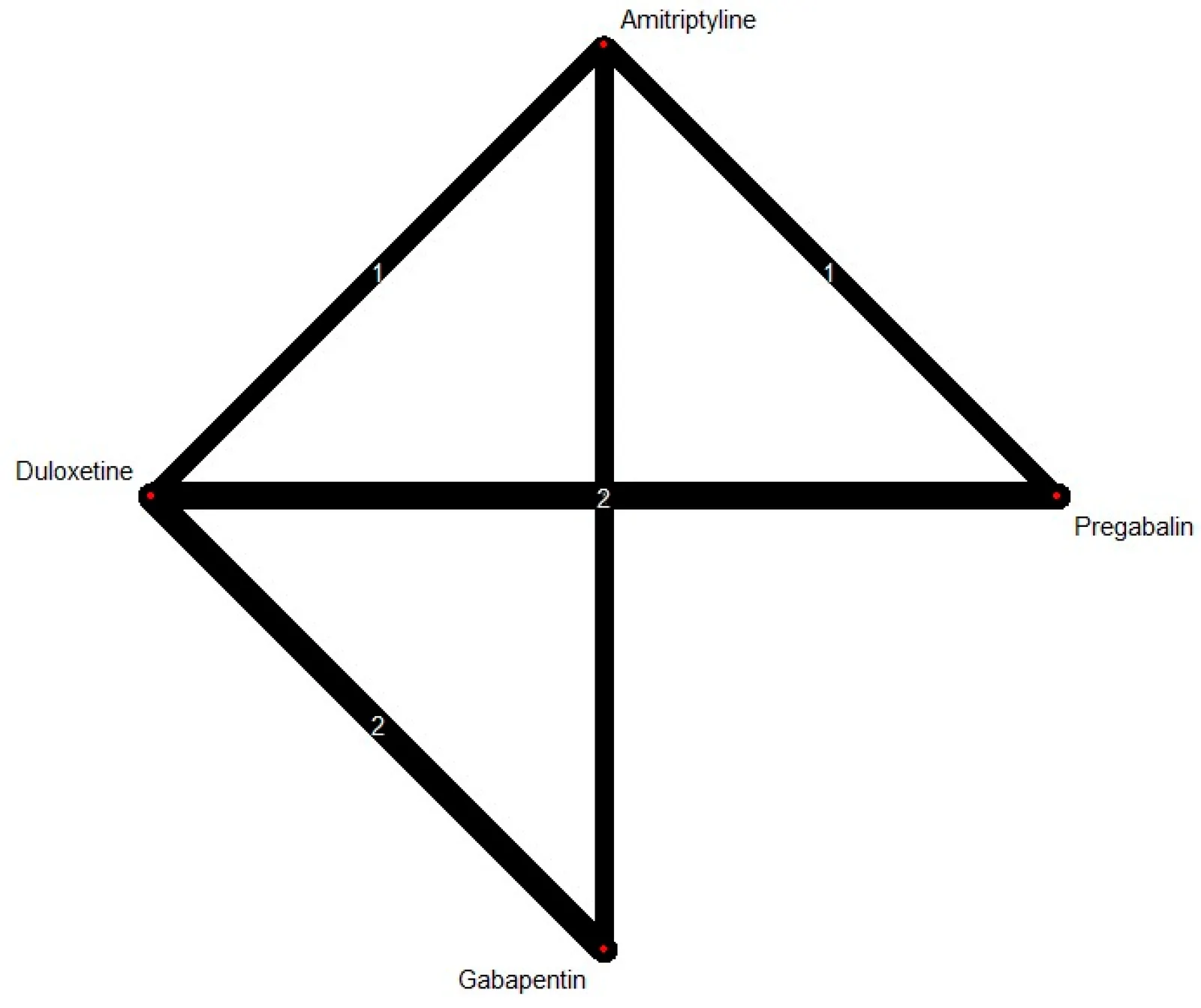
Network plot of direct treatment comparisons. Nodes represent the evaluated pharmacological agents. Edges (lines) connect treatments that have been directly compared in randomized controlled trials, with numbers on the edges indicating the number of studies (N) contributing to that specific comparison.

**Figure 4:**
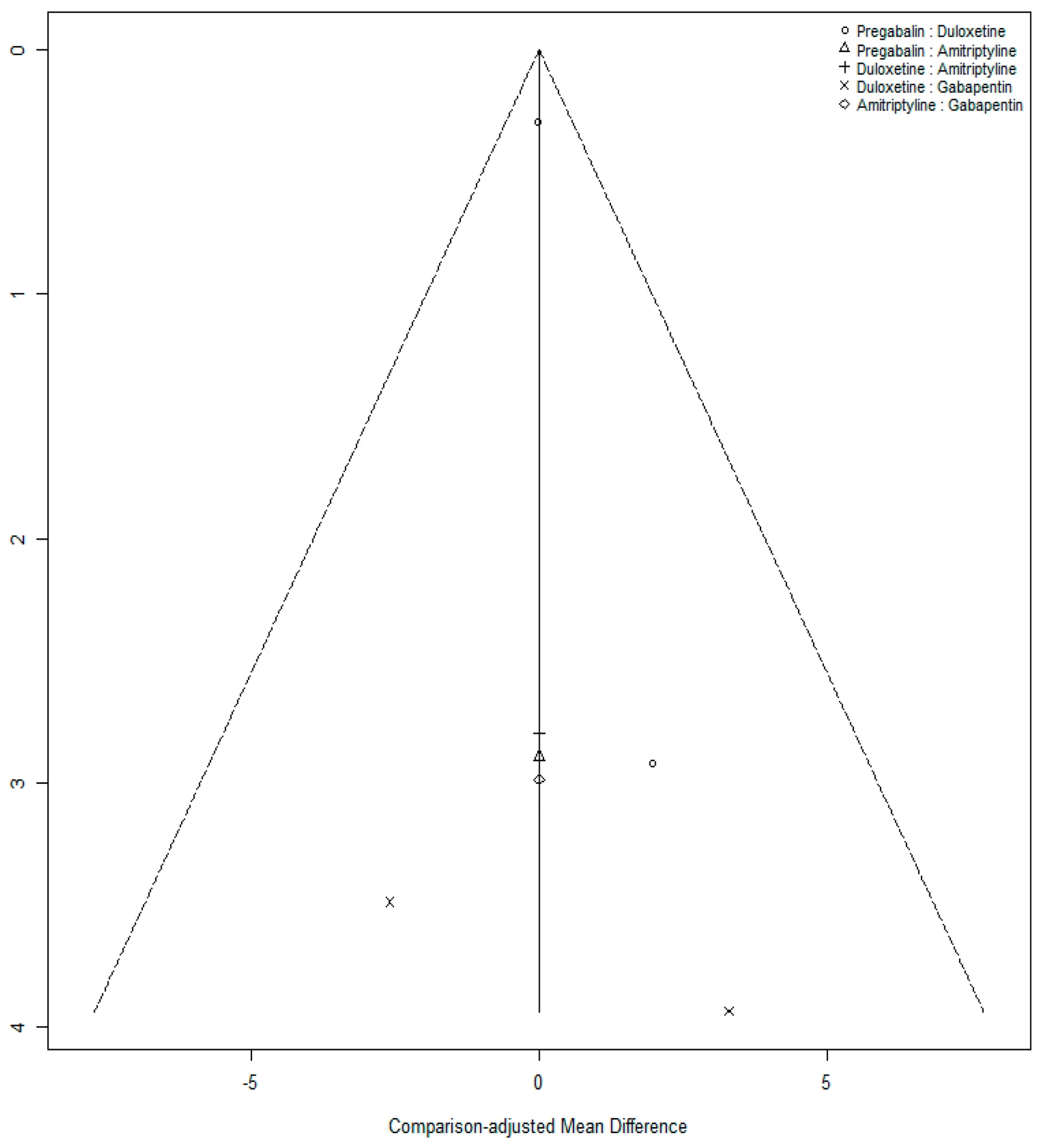
Comparison-adjusted funnel plot for the network meta-analysis under a moderate pre-post correlation assumption (ρ = 0.5). Each point represents an individual study plotted by its comparison-adjusted mean difference and standard error. The vertical line denotes the pooled comparison-adjusted effect, and the triangular region indicates the expected distribution in the absence of small-study effects.

**Table 1:** Characteristics of studies included in meta-analysis.

Study (Author, Year)	Drug	Dose (mg)	Duration (Weeks)	Sample Size (n)	Pain Scale	Key Outcome
Tesfaye et al. (2022) [20]	Amitriptyline	56	6	104	NRS	Significant pain reduction vs. baseline for all 3
	Duloxetine	76	6	100	NRS	No significant difference between the 3 agents
	Pregabalin	397	6	107	NRS	
Srinivasan et al. (2021) [21]	Amitriptyline	10–50	6	67	VAS	Significant pain reduction vs. baseline
Reddy et al. (2025) [22]	Amitriptyline	50	12	30	NRS	Significant pain reduction vs. baseline
	Gabapentin	1800	12	30	NRS	No significant difference between the 2 agents
Khasbage et al. (2021) [23]	Duloxetine	60	12	43	VAS	Significant pain reduction vs. baseline
	Gabapentin	300	12	43	VAS	No significant difference between the 2 agents
Majdinasab et al. (2019) [24]	Duloxetine	60	8	47	VAS	Significant pain reduction vs. baseline
	Gabapentin	900	8	41	VAS	No significant difference between the 2 agents
Gao et al. (2015) [25]	Duloxetine	60	12	173	BPI-Severity	Significant pain reduction vs. baseline
Yasuda et al. (2016) [26]	Duloxetine	60	52	129	BPI-Severity	Significant pain reduction vs. baseline
Tanenberg et al. (2011) [27]	Duloxetine	60	12	87	BPI-Severity	Significant pain reduction vs. baseline
	Pregabalin	300	12	99	BPI-Severity	No significant difference between the 2 agents
Rauck et al. (2013) [28]	Pregabalin	300	12	66	NRS	Significant pain reduction vs. placebo
Hassanzadeh et al. (2023) [29]	Gabapentin	100	8	15	VAS	Significant pain reduction vs. baseline
Ko et al. (2010) [30]	Gabapentin	1575	6	67	BPI-Severity	Significant pain reduction vs. baseline

NRS, Numeric Rating Scale; VAS, Visual Analog Scale; BPI, Brief Pain Severity.

**Table 2:** Pooled baseline demographic and clinical characteristics by pharmacologic agent.

Drug	No. of Studies (N)	Sample Size (n)	Age in Years (Mean ± SD)	Duration of Neuropathy in Years (Mean)	Mean HbA1c (%)	Percentage of Females
Duloxetine	6	665	60.47 ± 9.86	3.99	7.79	40.1
Pregabalin	3	307	60.96 ± 10.88	4.52	7.78	39.3
Gabapentin	5	224	58.11 ± 8.87	2.32	8.06	51.9
Amitriptyline	3	201	57.58 ± 10.31	3.83	8.04	35.5

**Table 3:** Relative treatment effects between all drug pairs from the network meta-analysis.

Comparison	Mean Difference	95% CI	*p*-Value
Amitriptyline vs. Pregabalin	5.76	1.66, 9.85	0.0059
Duloxetine vs. Pregabalin	4.98	4.40, 5.55	<0.0001
Gabapentin vs. Pregabalin	6.82	2.55, 11.08	0.0017
Amitriptyline vs. Duloxetine	0.78	−3.30, 4.86	0.71
Amitriptyline vs. Gabapentin	−1.06	−5.55, 3.44	0.64
Gabapentin vs. Duloxetine	1.84	−2.40, 6.08	0.39

CI, Confidence Interval.

**Table 4:** Sensitivity analysis of network meta-analysis results under low- and high-correlation assumptions.

	Low Correlation			High Correlation		
Comparison	Mean Difference	95% CI	*p*-Value	Mean Difference	95% CI	*p*-Value
Amitriptyline vs. Pregabalin	5.81	1.60, 10.03	0.0069	5.36	1.59, 9.13	0.0053
Duloxetine vs. Pregabalin	4.97	4.40, 5.55	<0.0001	4.98	4.41, 5.55	<0.0001
Amitriptyline vs. Duloxetine	0.84	−3.37, 5.04	0.696	0.38	−3.36, 4.13	0.8407
Amitriptyline vs. Gabapentin	−1.49	−6.45, 3.48	0.5572	−0.33	−4.10, 3.45	0.865
Gabapentin vs. Pregabalin	7.3	2.61, 11.99	0.0023	5.69	2.54, 8.84	0.0004
Gabapentin vs. Duloxetine	2.32	−2.34, 6.99	0.3287	0.71	−2.39, 3.82	0.653

CI, Confidence Interval.

## Data Availability

The original contributions presented in this study are included in the article/Supplementary Materials. Further inquiries can be directed to the corresponding author.

## Supplementary Materials

The following supporting information can be downloaded at: https://www.mdpi.com/article/10.3390/medsci14060649/s1. Figure S1: Funnel plot with trim-and-fill adjustment assessing the potential impact of publication bias on the pooled mean difference. The vertical dashed line represents the original pooled estimate, while the funnel region reflects the expected distribution under no small-study effects; Table S1: Risk-of-bias assessment of included studies; Table S2: Relative treatment effects between all drug pairs from the network meta-analysis for the sensitivity analysis (Unified 0–10 Scale, *ρ* = 0.5); Table S3: Subgroup Sensitivity Analysis Restricting the Network Meta-Analysis to Therapeutic Target Doses (Gabapentin ≥ 900 mg, *ρ* = 0.5); Table S4: Subgroup Sensitivity Analysis Restricting the Network Exclusively to Unidimensional Pain Scales (VAS/NRS); Table S5: Node-Splitting Validation (SIDE Approach) for Direct vs. Indirect Inconsistency; Table S6: Diabetes type reporting and stratified outcomes among included studies; Table S7: Comprehensive Within-Study Treatment Contrasts Used as Input Dataset for Network Meta-Analysis (*ρ* = 0.5); PRISMA 2020 checklist.

## Abbreviations

DPN	Diabetic painful neuropathy
NRS	Numeric rating scale
VAS	Visual analog scale
BPI	Brief pain inventory
TCA	Tricyclic antidepressant
SNRI	Serotonin–norepinephrine reuptake inhibitor
