# Supplementary material for: Comparative Effectiveness of Tricyclic Antidepressants, Serotonin–Norepinephrine Reuptake Inhibitors, and Anticonvulsants for Diabetic Painful Neuropathy: A Systematic Review and Meta-Analysis
Source: Med Sci (Basel). 2026 Oct 8;14(6):649. doi: 10.3390/medsci14060649 (PMC13706783; doi:10.3390/medsci14060649)
Supplement: Supplementary file 1 [file medsci-14-00649-s001.zip › medsci-4513282-supplementary.pdf]

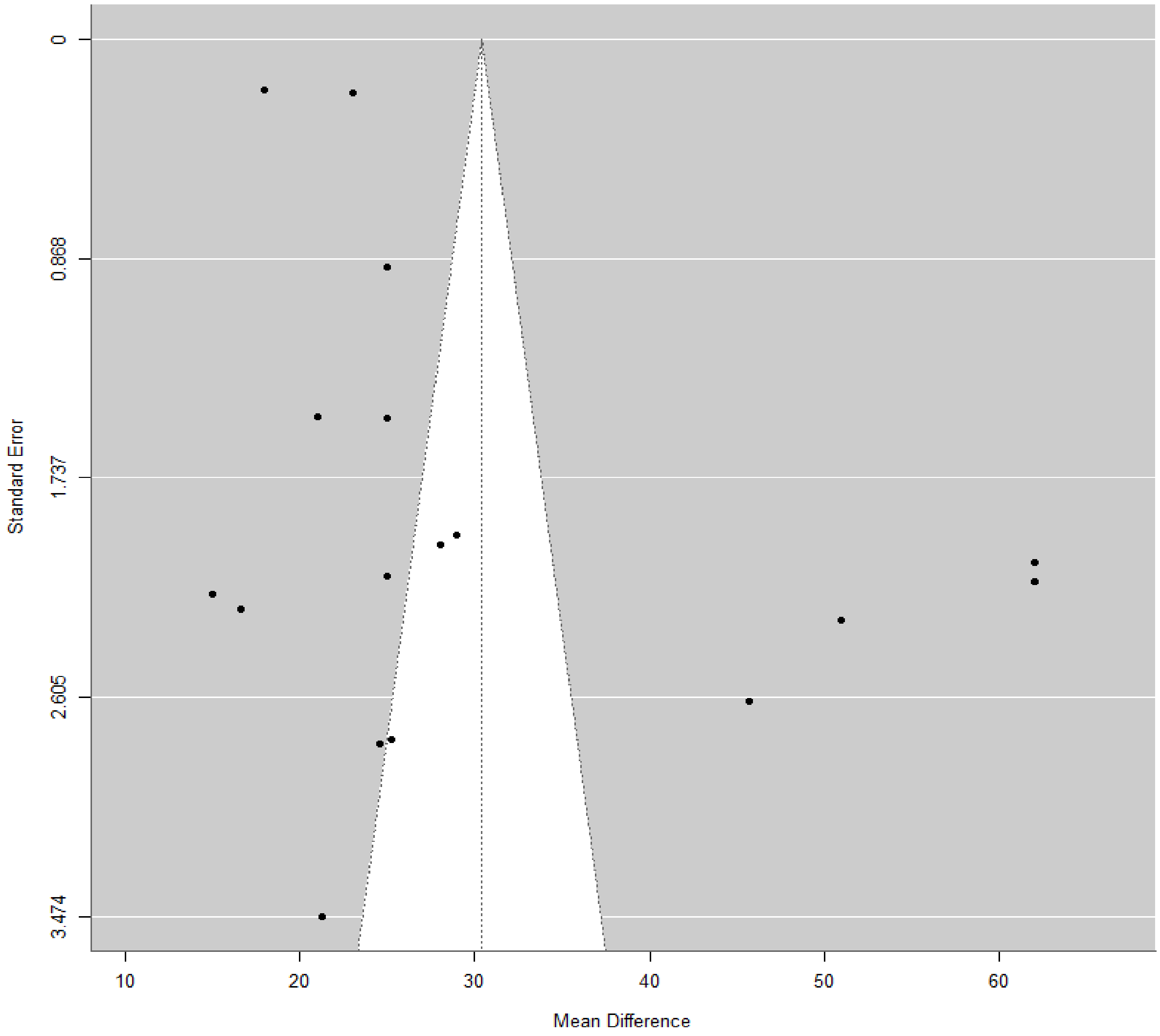

Supplementary Figure S1. *Funnel plot with trim-and-fill adjustment assessing the potential impact of publication bias on the pooled mean difference. The vertical dashed line represents the original pooled estimate, while the funnel region reflects the expected distribution under no small-study effects*

Supplementary Table S1. Risk-of-bias assessment of included studies

Study	D1	D2	D3	D4	D5	Overall	Brief rationale
**Gao, Y. et al., 2015**	Low	Low	Low	Low	Low	Low	Multicenter randomized double-blind placebo-controlled trial; ITT/MMRM; attrition reported/balanced.
**Hassanzadeh, S. et al., 2023**	Some concerns	Some concerns	Some concerns	Some concerns	Some concerns	Some concerns	Randomization/blinding and missing data handling incompletely described; subjective VAS outcome.
**Khasbage, S. et al., 2021**	Low	Some concerns	Low	Some concerns	Low	Some concerns	Computer block randomization and ITT/LOCF; open-label design for subjective pain outcome.
**Ko, S. et al., 2010**	Low	Some concerns	Some concerns	Some concerns	Some concerns	Some concerns	Computer-generated block randomization; open label; ~25% dropout; no clearly reported protocol/registration.
**Majdinasab, N. et al., 2019**	Some concerns	Low	Some concerns	Low	Low	Some concerns	Double-blind randomized registered trial; allocation-concealment and discontinuation handling not fully detailed.
**Rauck, R. et al., 2012**	Low	Low	Low	Low	Low	Low	Multicenter randomized double-blind double-dummy placebo-controlled trial; registered; prespecified PI-NRS endpoint.
**Reddy, S. et al., 2025**	Some concerns	High	Some concerns	High	Some concerns	High	Computer randomization reported, but open-label subjective outcome; limited concealment/missing-data/protocol details.
**Srinivasan, A. et al., 2021**	Low	Low	Low	Low	Low	Low	Random allocation software, concealment, blinding, ITT/PP analyses, and multiple imputation reported.
**Tanenberg, S. et al; 2011**	Some concerns	Some concerns	Some concerns	Some concerns	Low	Some concerns	Registered randomized noninferiority trial; open label with subjective diary pain outcome and substantial discontinuation.
**Tesfaye S. et al., 2022**	Low	Low	Low	Low	Low	Low	Double-blind crossover trial; stratified permuted-block randomization; protocol/registration; missing-data sensitivity analyses.
**Yasuda, H. et al., 2016**	Some concerns	Some concerns	Some concerns	Some concerns	Low	Some concerns	Randomized registered open-label extension of prior trial; prior-trial completer selection and discontinuations.

Domain definitions: D1 = randomization process; D2 = deviations from intended interventions; D3 = missing outcome data; D4 = measurement of outcome; D5 = selection of reported result.

Supplementary Table S2. Relative treatment effects between all drug pairs from the network meta-analysis for the sensitivity analysis (Unified 0–10 Scale, $\rho = 0.5$).

Comparison	Mean Difference (MD)	95% Confidence Interval (CI)	\(P\)-value
**Amitriptyline vs. Pregabalin**	0.58	[0.17; 0.99]	0.0059
**Duloxetine vs. Pregabalin**	0.50	[0.44; 0.55]	< 0.0001
**Gabapentin vs. Pregabalin**	0.68	[0.25; 1.11]	0.0017
**Duloxetine vs. Amitriptyline**	-0.08	[-0.49; 0.33]	0.7076
**Gabapentin vs. Amitriptyline**	0.11	[-0.34; 0.56]	0.6443
**Gabapentin vs. Duloxetine**	0.18	[-0.24; 0.61]	0.3949

(Note: Point estimates represent absolute pain reductions on a unified 0–10 range. The directional hierarchy, z-scores, and p-values perfectly match the primary 0–100 scale model, confirming extreme network stability.)

Supplementary Table S3. Subgroup Sensitivity Analysis Restricting the Network Meta-Analysis to Therapeutic Target Doses (Gabapentin ≥ 900 mg, $\rho = 0.5$).

Baseline Reference	Comparison Treatment	Mean Difference (MD)	95% Confidence Interval (CI)	\(P\)-value
**Pregabalin Baseline**	Amitriptyline	4.91	[0.60; 9.22]	0.0255
	Duloxetine	4.98	[4.41; 5.55]	< 0.0001
	Gabapentin	4.70	[-0.72; 10.12]	0.0892
**Duloxetine Baseline**	Amitriptyline	-0.07	[-4.37; 4.23]	0.9741
	Gabapentin	-0.28	[-5.68; 5.12]	0.9183
	Pregabalin	-4.98	[-5.55; -4.41]	< 0.0001
**Gabapentin Baseline**	Amitriptyline	0.21	[-4.71; 5.13]	0.9328
	Duloxetine	0.28	[-5.12; 5.68]	0.9183
	Pregabalin	-4.70	[-10.12; 0.72]	0.0892

Supplementary Table S4. Subgroup Sensitivity Analysis Restricting the Network Exclusively to Unidimensional Pain Scales (VAS/NRS)

Baseline Reference	Comparison Treatment	Mean Difference (MD)	95% Confidence Interval (CI)	P-value	Status vs. Main Model
**Pregabalin Baseline**	Amitriptyline	4.27	[-1.18; 9.72]	0.1250	Non-Significant
	Duloxetine	2.72	[-2.77; 8.21]	0.3314	Non-Significant
	Gabapentin	4.89	[-1.42; 11.21]	0.1288	Non-Significant
**Duloxetine Baseline**	Amitriptyline	1.55	[-2.94; 6.03]	0.4986	Non-Significant
	Gabapentin	2.17	[-2.14; 6.49]	0.3238	Non-Significant
	Pregabalin	-2.72	[-8.21; 2.77]	0.3314	Non-Significant
**Gabapentin Baseline**	Amitriptyline	-0.62	[-5.24; 3.99]	0.7912	Non-Significant
	Duloxetine	-2.17	[-6.49; 2.14]	0.3238	Non-Significant
	Pregabalin	-4.89	[-11.21; 1.42]	0.1288	Non-Significant

Abbreviations: CI, confidence interval; MD, mean difference; NRS, numeric rating scale; VAS, visual analog scale. Note: Mean differences represent absolute pain reduction on the harmonized 0–100 scale. A value greater than zero indicates a numerical advantage over the baseline reference group.

Supplementary Table S5: Node-Splitting Validation (SIDE Approach) for Direct vs. Indirect Inconsistency

Comparison Node Pathway	Direct Studies (n)	Direct Proportion	Network MD	Direct MD	Indirect MD	Inconsistency P-value
**Amitriptyline vs. Duloxetine**	1	0.55	0.78	1.00	0.51	**0.9071**
**Amitriptyline vs. Gabapentin**	1	0.59	-1.06	0.00	-2.58	**0.5802**
**Amitriptyline vs. Pregabalin**	1	0.52	5.76	4.00	7.67	**0.3801**
**Duloxetine vs. Gabapentin**	2	0.69	-1.84	-2.65	-0.07	**0.5802**
**Duloxetine vs. Pregabalin**	2	1.00	4.98	4.98	-0.51	**0.5802**

(Note: The uniformly high p-values across all evaluated loops mathematically confirm that there is no statistically significant disagreement between the direct trial evidence and the indirect network calculations, validating the internal consistency of the global estimates.)

Supplementary Table S6. Diabetes type reporting and stratified outcomes among included studies

Study	Type 1 diabetes (%)	Type 2 diabetes (%)	Study population diabetes type reported?	Stratified efficacy outcomes by diabetes type reported?
**Ko et al. 2010**	0	100	Yes; Type 2 diabetes only	No
**Tanenberg et al. 2011**	Not reported	Not reported	Type 1 or Type 2 eligible, but exact distribution not reported	No
**Rauck et al. 2012**	Not reported	Not reported	Type 1 or Type 2 eligible, but exact distribution not reported	No
**Gao et al. 2015**	1.5	98.5	Yes	No
**Yasuda et al. 2016**	4.7	95.3	Yes	No
**Majdinasab et al. 2019**	Not reported	Not reported	No; diabetes type not clearly specified	No
**Khasbage et al. 2021**	0	100	Yes; Type 2 diabetes only	No
**Srinivasan et al. 2021**	0	100	Yes; Type 2 diabetes only	No
**Tesfaye et al. 2022**	17.0	81.5	Yes	No
**Hassanzadeh et al. 2023**	0	100	Yes; Type 2 diabetes only	No
**Reddy et al. 2025**	Not reported	Not reported	No; diabetes type not clearly specified	No

Supplementary Table S7: Comprehensive Within-Study Treatment Contrasts Used as Input Dataset for Network Meta-Analysis (ρ=0.5)

Primary Study	Treatment 1 (Active)	Treatment 2 (Comparator)	Mean Difference (MD)	Standard Error (SE)	Sample Size (N1)	Sample Size (N2)
**Khasbage et al. (2021)**	Duloxetine	Gabapentin	-5.20	3.490	43	43
**Majdinasab N et al. (2019)**	Duloxetine	Gabapentin	0.60	3.936	47	41
**Reddy et al. (2025)**	Amitriptyline	Gabapentin	0.00	2.996	30	30
**Tanenberg et al. (2011)**	Duloxetine	Pregabalin	5.00	0.294	87	99
**Tesfaye et al. (2022)**	Amitriptyline	Duloxetine	1.00	2.801	104	100
**Tesfaye et al. (2022)**	Amitriptyline	Pregabalin	4.00	2.893	104	107
**Tesfaye et al. (2022)**	Duloxetine	Pregabalin	3.00	2.919	100	107

PRISMA 2020 Checklist

Section and Topic	Item #	Checklist item	Location where item is reported
**TITLE**			
Title	1	Identify the report as a systematic review.	Title page
**ABSTRACT**			
Abstract	2	See the PRISMA 2020 for Abstracts checklist.	Abstract, Page #1-2
**INTRODUCTION**			
Rationale	3	Describe the rationale for the review in the context of existing knowledge.	Introduction, Page #2
Objectives	4	Provide an explicit statement of the objective(s) or question(s) the review addresses.	Introduction, Page #2
**METHODS**			
Eligibility criteria	5	Specify the inclusion and exclusion criteria for the review and how studies were grouped for the syntheses.	Materials and Methods: Section 2.2. Inclusion and Exclusion criteria, Page #3
Information sources	6	Specify all databases, registers, websites, organisations, reference lists and other sources searched or consulted to identify studies. Specify the date when each source was last searched or consulted.	Materials and Methods: Section 2.3. Data extraction and search strategy, Page #3-7
Search strategy	7	Present the full search strategies for all databases, registers and websites, including any filters and limits used.	Materials and Methods: Section 2.1. Data source and search strategy, Page #3-7
Selection process	8	Specify the methods used to decide whether a study met the inclusion criteria of the review, including how many reviewers screened each record and each report retrieved, whether they worked independently, and if applicable, details of automation tools used in the process.	Materials and Methods: Data source & search strategy & Data extraction and synthesis, Page #3-7 and Figure 1.
Data collection process	9	Specify the methods used to collect data from reports, including how many reviewers collected data from each report, whether they worked independently, any processes for obtaining or confirming data from study investigators, and if applicable, details of automation tools used in	Materials and Methods: Data

PRISMA 2020 Checklist

Section and Topic	Item #	Checklist item	Location where item is reported
		the process.	source & search strategy & Data extraction and synthesis, Page #3-7
Data items	10a	List and define all outcomes for which data were sought. Specify whether all results that were compatible with each outcome domain in each study were sought (e.g. for all measures, time points, analyses), and if not, the methods used to decide which results to collect.	Methods: Inclusion criteria / Data extraction and synthesis, Page #4-5
	10b	List and define all other variables for which data were sought (e.g. participant and intervention characteristics, funding sources). Describe any assumptions made about any missing or unclear information.	Methods: Data extraction and synthesis, Page #5-6
Study risk of bias assessment	11	Specify the methods used to assess risk of bias in the included studies, including details of the tool(s) used, how many reviewers assessed each study and whether they worked independently, and if applicable, details of automation tools used in the process.	Methods: Risk of bias assessment, Page #5, Supplementary Table 1
Effect measures	12	Specify for each outcome the effect measure(s) (e.g. risk ratio, mean difference) used in the synthesis or presentation of results.	Data extraction and synthesis, Page #3-4
Synthesis methods	13a	Describe the processes used to decide which studies were eligible for each synthesis (e.g. tabulating the study intervention characteristics and comparing against the planned groups for each synthesis (item #5)).	Figure 1, Table 1
	13b	Describe any methods required to prepare the data for presentation or synthesis, such as handling of missing summary statistics, or data conversions.	Methods: Data extraction and synthesis, Page #3-4
	13c	Describe any methods used to tabulate or visually display results of individual studies and syntheses.	Statistical Analysis, Page 4-5
	13d	Describe any methods used to synthesize results and provide a rationale for the choice(s). If meta-analysis was performed, describe the model(s), method(s) to identify the presence and extent of statistical heterogeneity, and software package(s) used.	Methods: Statistical Analysis, Page #4-5
	13e	Describe any methods used to explore possible causes of heterogeneity among study results (e.g. subgroup analysis, meta-regression).	Methods: Statistical Analysis, Page #4-5 Limitations,

PRISMA 2020 Checklist

Section and Topic	Item #	Checklist item	Location where item is reported
			Page # 16-17
	13f	Describe any sensitivity analyses conducted to assess robustness of the synthesized results.	Methods: Statistical Analysis, Page #5-6
Reporting bias assessment	14	Describe any methods used to assess risk of bias due to missing results in a synthesis (arising from reporting biases).	Methods: Publication Bias, Page #5
Certainty assessment	15	Describe any methods used to assess certainty (or confidence) in the body of evidence for an outcome.	Methods: Statistical Analysis, page 4-5
**RESULTS**			
Study selection	16a	Describe the results of the search and selection process, from the number of records identified in the search to the number of studies included in the review, ideally using a flow diagram.	Figure 1 (Page #6), Results: Search results, Page #7
	16b	Cite studies that might appear to meet the inclusion criteria, but which were excluded, and explain why they were excluded.	Missing
Study characteristics	17	Cite each included study and present its characteristics.	Results: Table 1, Page #7-8
Risk of bias in studies	18	Present assessments of risk of bias for each included study.	Methods, Risk of Bias, page 4
Results of individual studies	19	For all outcomes, present, for each study: (a) summary statistics for each group (where appropriate) and (b) an effect estimate and its precision (e.g. confidence/credible interval), ideally using structured tables or plots.	Results: Table 1 and Figure 2
Results of syntheses	20a	For each synthesis, briefly summarise the characteristics and risk of bias among contributing studies.	Results: Study characteristics, Page #7-8; risk of bias Page 4
	20b	Present results of all statistical syntheses conducted. If meta-analysis was done, present for each the summary estimate and its precision (e.g. confidence/credible interval) and measures of statistical heterogeneity. If comparing groups, describe the direction of the effect.	Results: Random-effects meta-analysis and Between-drug comparisons, Page #8-11
	20c	Present results of all investigations of possible causes of heterogeneity among study results.	Results: Between-drug comparisons / heterogeneity paragraph, Page #10-11,

Section and Topic	Item #	Checklist item	Location where item is reported
			15-17
	20d	Present results of all sensitivity analyses conducted to assess the robustness of the synthesized results.	Results: Sensitivity analyses, Table 4
Reporting biases	21	Present assessments of risk of bias due to missing results (arising from reporting biases) for each synthesis assessed.	Results: Assessment of Publication Bias and Small-Study Effects, Page #14; Figure 3, Supplementary Figure S1
Certainty of evidence	22	Present assessments of certainty (or confidence) in the body of evidence for each outcome assessed.	Missing
**DISCUSSION**			
Discussion	23a	Provide a general interpretation of the results in the context of other evidence.	Discussion: Current Treatment Landscape and Prior Evidence, Page #15-16
	23b	Discuss any limitations of the evidence included in the review.	Discussion: Limitations, Page #17
	23c	Discuss any limitations of the review processes used.	Discussion: Limitations, Page #17
	23d	Discuss implications of the results for practice, policy, and future research.	Discussion: Implications for Future Research. Conclusion, Page #18
**OTHER INFORMATION**			
Registration and protocol	24a	Provide registration information for the review, including register name and registration number, or state that the review was not registered.	Statements & Declarations: Registration and Protocol, Page #18

PRISMA 2020 Checklist

Section and Topic	Item #	Checklist item	Location where item is reported
	24b	Indicate where the review protocol can be accessed, or state that a protocol was not prepared.	Statements & Declarations: Registration and Protocol, Page #7
	24c	Describe and explain any amendments to information provided at registration or in the protocol.	Statements & Declarations: Registration and Protocol, Page #7
Support	25	Describe sources of financial or non-financial support for the review, and the role of the funders or sponsors in the review.	Statements & Declarations: Funding, Page #18
Competing interests	26	Declare any competing interests of review authors.	Statements & Declarations: Competing interests, Page #18
Availability of data, code and other materials	27	Report which of the following are publicly available and where they can be found: template data collection forms; data extracted from included studies; data used for all analyses; analytic code; any other materials used in the review.	Supplementary Materials

From: Page MJ, McKenzie JE, Bossuyt PM, Boutron I, Hoffmann TC, Mulrow CD, et al. The PRISMA 2020 statement: an updated guideline for reporting systematic reviews. BMJ 2021;372:n71. doi: 10.1136/bmj.n71. This work is licensed under CC BY 4.0. To view a copy of this license, visit https://creativecommons.org/licenses/by/4.0/
